# MKL1 promotes endothelial-to-mesenchymal transition and liver fibrosis by activating TWIST1 transcription

**DOI:** 10.1038/s41419-019-2101-4

**Published:** 2019-11-27

**Authors:** Zilong Li, Baoyu Chen, Wenhui Dong, Ming Kong, Zhiwen Fan, Liming Yu, Dongmei Wu, Jun Lu, Yong Xu

**Affiliations:** 1 Key Laboratory of Targeted Intervention of Cardiovascular Disease and Collaborative Innovation Center for Cardiovascular Translational Medicine, Department of Pathophysiology, Nanjing Medical University, Nanjing, China; 2 Institute of Biomedical Research, Liaocheng University, Liaocheng, China; 3 Key Laboratory of Biotechnology on Medical Plants of Jiangsu Province and School of Life Sciences, Jiangsu Normal University, Xuzhou, China; 4 College of Health Sciences, Jiangsu Normal University, Xuzhou, China

**Keywords:** Transcription, Liver fibrosis

## Abstract

Excessive fibrogenic response in the liver disrupts normal hepatic anatomy and function heralding such end-stage liver diseases as hepatocellular carcinoma and cirrhosis. Sinusoidal endothelial cells contribute to myofibroblast activation and liver fibrosis by undergoing endothelial-mesenchymal transition (EndMT). The underlying mechanism remains poorly defined. Here we report that inhibition or endothelial-specific deletion of MKL1, a transcriptional modulator, attenuated liver fibrosis in mice. MKL1 inhibition or deletion suppressed EndMT induced by TGF-β. Mechanistically, MKL1 was recruited to the promoter region of TWIST1, a master regulator of EndMT, and activated TWIST1 transcription in a STAT3-dependent manner. A small-molecule STAT3 inhibitor (C188-9) alleviated EndMT in cultured cells and bile duct ligation (BDL) induced liver fibrosis in mice. Finally, direct inhibition of TWIST1 by a small-molecule compound harmine was paralleled by blockade of EndMT in cultured cells and liver fibrosis in mice. In conclusion, our data unveil a novel mechanism underlying EndMT and liver fibrosis and highlight the possibility of targeting the STAT3-MKL1-TWIST1 axis in the intervention of aberrant liver fibrogenesis.

## Introduction

Liver fibrosis is generally perceived as a host defense mechanism following liver injury. Secondary to trauma, uptake of excessive nutrition, infection of pathogens, and exposure to hepatotoxic substances, fibrogenesis reins in liver damage, facilitates wound closure, and safeguards the structural and functional integrity of the liver. Uncontrolled and/or unresolved liver fibrosis, however, serves to disrupt normal liver structure, is associated with loss of critical key liver functions, and heralds such end-stage liver diseases as hepatocellular carcinoma and cirrhosis^1,2^. Regardless of its etiology, liver fibrosis is believed to be mediated by the activation of myofibroblasts, which possess the ability to contract and thus cover the wound and the capability of producing extracellular matrix (ECM) proteins^3^. It remains controversial as to where the population of activated myofibroblasts originates from during liver fibrosis. Many have considered hepatic stellate cells (HSCs) as the major source of ECM producing myofibroblasts, a notion that has been both validated and challenged by genetic lineage tracing experiments^4,5^. Biliary portal fibroblast cells^4^, hepatocytes^6^, and bone marrow-derived fibrocyte^7^ have also been found to contribute to the pool of fibrogenic myofibroblasts in the liver. In addition, recent investigations have led to the revelation that liver sinusoidal endothelial cells may give rise to activated myofibroblasts via a process known as endothelial-to-mesenchymal transition, or EndMT^8,9^.

EndMT is a developmentally pivotal process key to organogenesis^10^. Post-embryonic EndMT more often than not correlates with the onset of a wide range of human diseases. During EndMT, endothelial cells switch from a cobblestone-like morphology to a spindle-like outlook and acquire augmented ability to migrate and produce ECM components. The morphological changes are paralleled by a profound alteration in gene expression profiles: endothelial signature genes (e.g., VE-Cadherin and CD31) are down-regulated in favor of mesenchymal specific genes (e.g., α-SMA and collagen type I)^11^. EndMT can be evoked by a host of factors, the most prominent among which is transforming growth factor (TGF-β)^12^. In the nucleus, TGF-β induced EndMT is programmed by a group of conserved transcription factors. On the one hand, TGF-β activates the Snail family of transcriptional repressors, including Snail, Slug, Twist, and Zeb, to repress the transcription of endothelial marker genes^13^. On the other hand, TGF-β promotes the activation of SMAD family of transcription activators, which in turn switch on the synthesis of mesenchymal markers^14^. Although EndMT has been identified as a contributing factor to liver fibrosis both in experimental animals^9^ and in humans^8^, the regulatory mechanism underlying TGF-β dependent EndMT and liver fibrosis remains incompletely appreciated.

Megakaryocytic leukemia 1 (MKL1) is a multifaceted transcriptional modulator implicated in the pathogenesis of an array of human diseases^15^. Mounting evidence points to an indispensable role for MKL1 in myofibroblast activation and, by extension, tissue fibrosis^16–19^. We have previously shown that systemic MKL1 knockout mice show attenuation of liver fibrosis owing to defective activation of HSCs^20,21^. Here we present evidence to show that MKL1 is essential for TGF-β induced EndMT in cultured cells by cooperating with the transcription factor STAT3 to activate TWIST1 transcription. Genetic deletion of MKL1 in endothelial cells or pharmaceutical inhibition of MKL1 or STAT3 or TWIST1 is sufficient to alleviate liver fibrosis in mice. Therefore, targeting the STAT3-MKL1-TWIST1 axis may yield novel therapeutic solutions against dysregulated liver fibrosis.

## Results

### Endothelial MKL1 deficiency attenuates liver fibrosis in mice

We have previously shown that germline deletion of MKL1 ameliorates liver fibrosis in mice^20,21^. In order to delineate cell-specific roles for MKL1 in liver fibrosis, we crossbred the *Mkl1*^f/f^ strain with a *Cdh5*-ERT2-Cre strain^22^ to restrictively delete MKL1 in vascular endothelial cells. As shown in Fig. S1, there were fewer CD31+ MKL1+ cells in the endothelial-specific MKL1 knockout (ecKO^m/m^) mice compared to the wild type (WT) mice, suggesting that MKL1 was indeed deleted from endothelial cells.

**Fig. 1:**
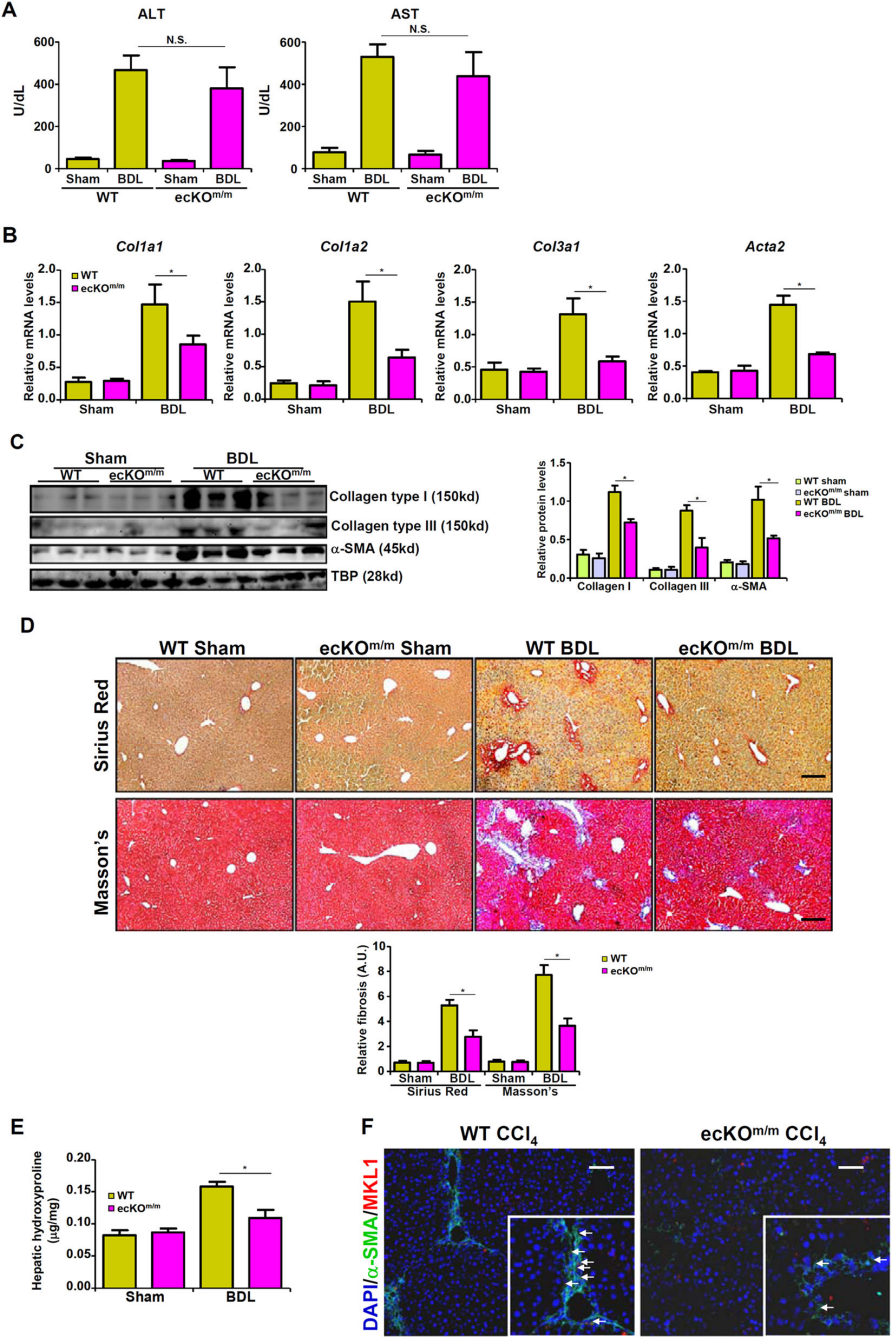
Endothelial MKL1 deficiency attenuates BDL-induced liver fibrosis in mice. Liver fibrosis was induced in WT or endothelial-specific MKL1 knockout (ecKO^m/m^) mice by BDL. **a** Plasma ALT levels and AST levels. Expression levels of pro-fibrogenic genes were examined by qPCR (**b**) and Western (**c**). **d** Picrosirius red and Masson’s trichrome stainings. **e** Hepatic hydroxylproline levels. **f** Immunofluorescence staining was performed with anti-MKL1 (red) and anti-α-SMA (green). *N* = 4 mice for the sham groups and *N* = 8 mice for the BDL groups.

Both WT and ecKO^m/m^ mice were subjected to bile duct ligation to induce liver fibrosis. Unlikely systemic MKL1 knockout mice that exhibited reduced liver injury^20,21^, the ecKO^m/m^ mice and the WT littermates showed comparable liver injuries as evidenced by plasma ALT and AST levels (Fig. 1a). Liver fibrosis, however, was significantly attenuated in ecKO^m/m^ mice, as demonstrated by qPCR (Fig. 1b) and Western (Fig. 1c) analyses of pro-fibrogenic gene expression levels. In addition, picrosirius red and Masson’s trichrome stainings also showed that liver fibrosis was less widespread in ecKO^m/m^ mice compared to WT mice (Fig. 1d). Quantitative measurements of hepatic hydroxylproline levels indicated that the production of fibrillar collagens was down-regulated in ecKO^m/m^ mice as opposed to WT mice (Fig. 1e). In addition, immunofluorescence staining confirmed that there were fewer MKL1^+^α-SMA^+^ cells in the ecKO^m/m^ mice than in the WT mice (Fig. 1f).

**Fig. 2:**
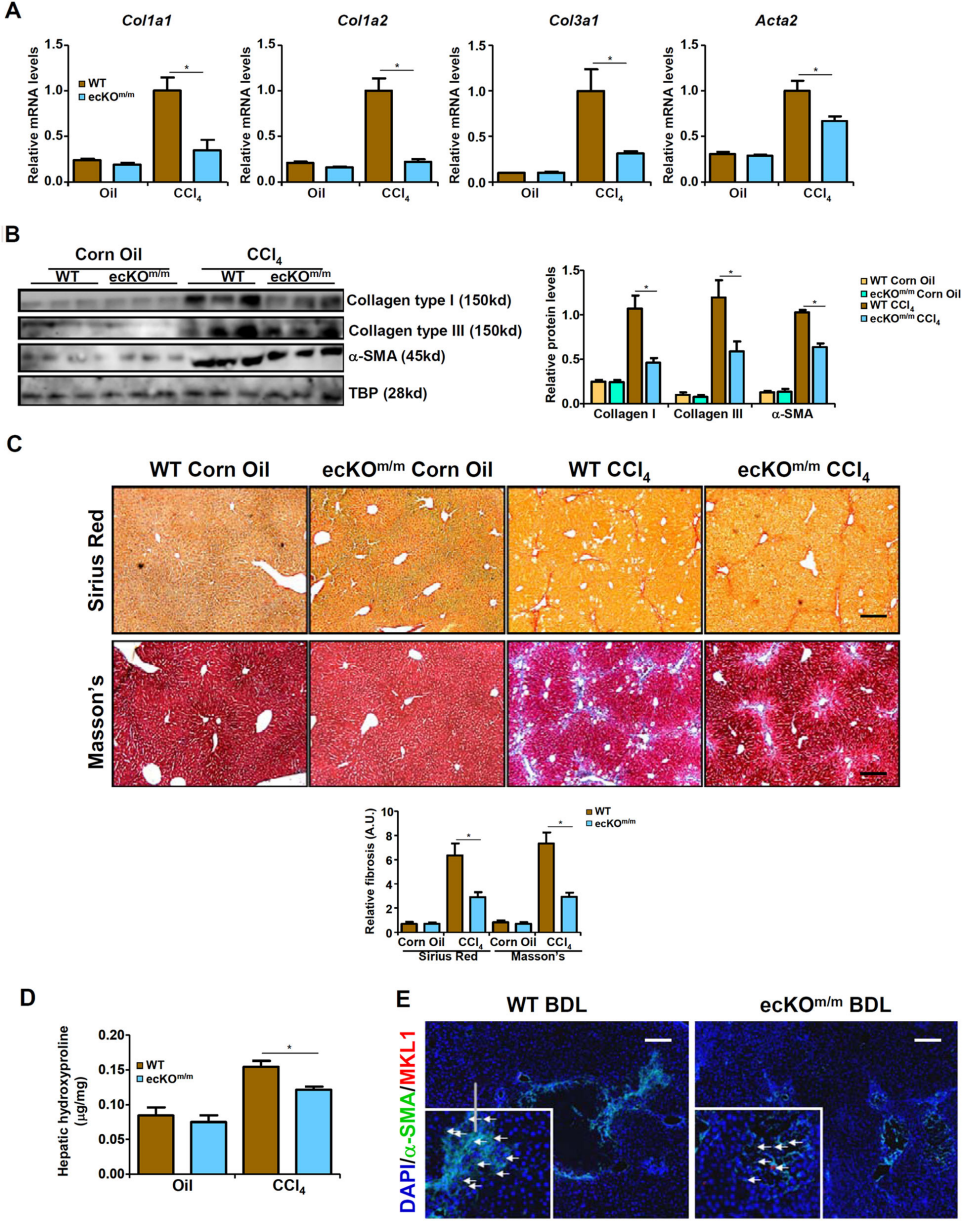
Endothelial MKL1 deficiency attenuates CCl_4_-induced liver fibrosis in mice. Liver fibrosis was induced in WT or endothelial-specific MKL1 knockout (ecKO^m/m^) mice by CCl_4_ injection. Expression levels of pro-fibrogenic genes were examined by qPCR (**a**) and western (**b**). **c** Picrosirius red and Masson’s trichrome stainings. **d** Hepatic hydroxylproline levels. **e** Immunofluorescence staining was performed with anti-MKL1 (red) and anti-α-SMA (green). *N* = 4 mice for the sham groups and *N* = 6 mice for the BDL groups.

We then attempted to validate these observations in a different model of liver fibrosis in which the mice were injected with CCl_4_ weekly for 6 weeks. Similar to the BDL model, CCl_4_ induced liver injuries were not significantly different between the ecKO^m/m^ mice and the WT mice (data not shown). Quantitative measurements of mRNA (Fig. 2a) and protein (Fig. 2b) levels of pro-fibrogenic gene expression indicated that liver fibrosis induced by CCl_4_ by down-regulated in ecKO^m/m^ mice compared to WT mice. Consistently, picrosirius red and Masson’s stainings (Fig. 2c) as well as hepatic hydroxylproline quantification (Fig. 2d) showed a decrease in liver fibrogenesis. Immunofluorescence staining further confirmed that MKL1^+^α-SMA^+^ cells were down-regulated in the ecKO^m/m^ mice compared to the WT mice (Fig. 1e). Combined, these data argue for an endothelial-specific role of MKL1 in liver fibrosis.

### MKL1 deficiency or inhibition suppresses EndMT

**Fig. 3:**
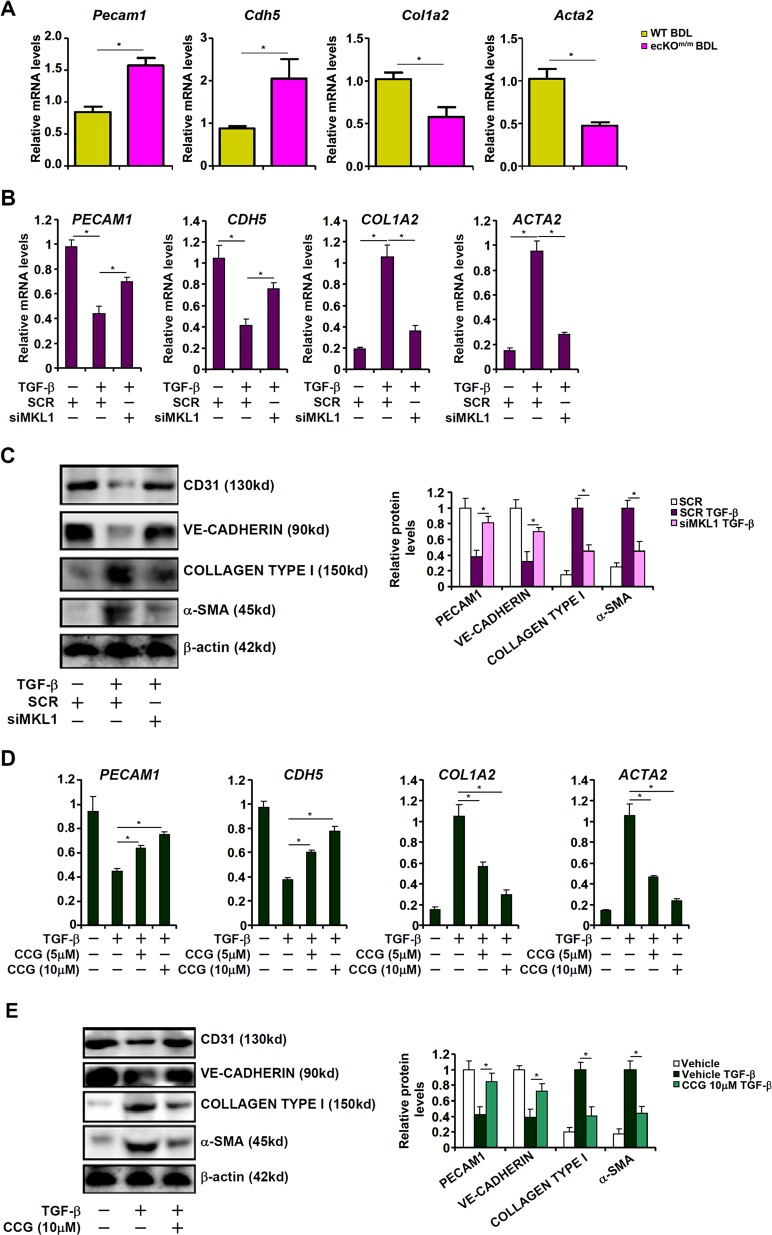
MKL1 deficiency or inhibition suppresses EndMT. **a** Liver fibrosis was induced in WT or ecKO^m/m^ mice by BDL. Primary sinusoidal endothelial cells were isolated and gene expression levels were examined by qPCR. **b**, **c** Human vascular endothelial cells were transfected with siRNA targeting MKL1 or scrambled siRNA (SCR) followed by treatment with TGF-β. Gene expression levels were examined by qPCR and western. **d**, **e** Human vascular endothelial cells were treated with TGF-β in the presence or absence of CCG-1423. Gene expression levels were examined by qPCR and western.

Next, we verified the hypothesis that MKL1 might regulate EndMT thereby contributing to liver fibrosis. Indeed, primary liver sinusoidal endothelial cells (LSECs) isolated from ecKO^m/m^ mice subjected to BDL expressed higher levels of *Cdh5* (encoding VE-Cadherin) and *Pecam1* (encoding CD31), two signature endothelial markers, than those isolated from WT BDL mice (Fig. 3a). On the contrary, ecKO^m/m^ LSECs expressed lower levels of *Col1a2* (encoding Collagen type I) and *Acta2* (encoding α-SMA), two prominent mesenchymal markers, compared to WT LSECs, suggesting that MKL1 deficiency in endothelial may alleviate EndMT in the context of liver fibrosis in vivo. We then exploited two strategies to examine the role of MKL1 in TGF-β induced EndMT in primary human vascular endothelial cells (HVECs). TGF-β treatment down-regulated *CDH5* and *PECAM1* while simultaneously up-regulating *COL1A2* and *ACTA2*; depletion of MKL1 with siRNA (Fig. S2A for knockdown efficiency), however, antagonized TGF-β induced EndMT by partially normalizing gene expression levels (Fig. 3b, c). A second pair of MKL1 siRNA achieved similar effects in HVECs (Fig. S3).

Alternatively, we used CCG-1423, a well-established MKL1 inhibitor^23,24^, to treat HVECs. CCG-1423 treatment attenuated TGF-β induced EndMT, as evidenced in gene expression patterns, in a dose-dependent manner (Fig. 3d, e). More importantly, CCG-1423 administration alleviated liver fibrosis induced by both BDL (Fig. S4A, B) and CCl_4_ (Fig. S5A, B) in mice. Of note, LSECs isolated from CCG-treated mice showed higher expression levels of endothelial markers and lower levels of mesenchymal markers compared to those isolated from the control mice in both the BDL model (Fig. S4C) and the CCl_4_ model (Fig. S5C). Together, these data suggest that MKL1 may function as a key regulator of EndMT in vitro and in vivo.

### MKL1 activates TWIST1 transcription in endothelial cells

**Fig. 4:**
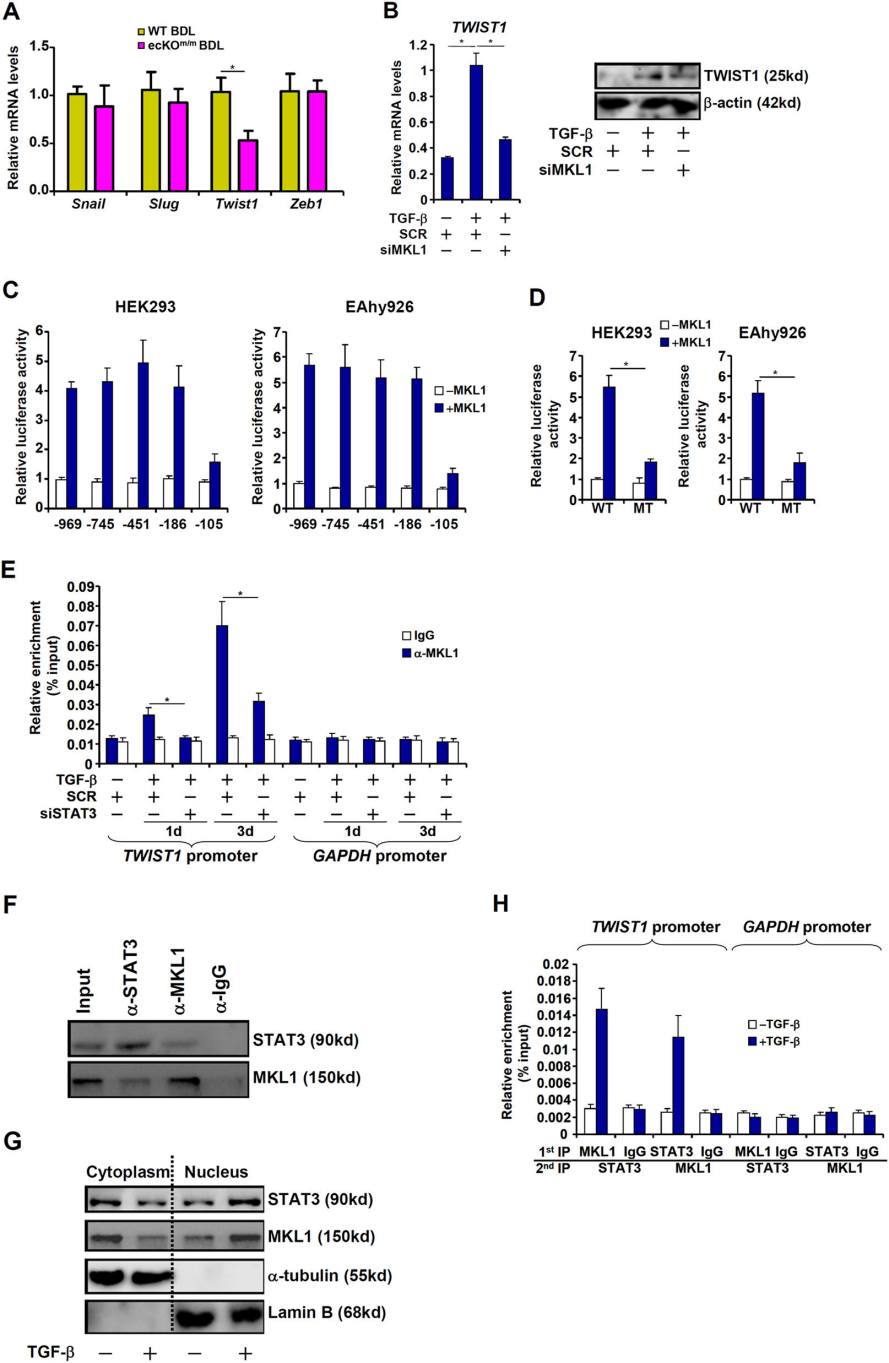
MKL1 activates TWIST1 transcription in endothelial cells. **a** Liver fibrosis was induced in WT or ecKO^m/m^ mice by BDL. Primary sinusoidal endothelial cells were isolated and gene expression levels were examined by qPCR. **b** Human vascular endothelial cells were transfected with siRNA targeting MKL1 or scrambled siRNA (SCR) followed by treatment with TGF-β. TWIST1 expression levels were examined by qPCR and western. **c** TWIST1 promoter-luciferase constructs were transfected with or without MKL1 into HEK293 cells or EAhy926 cells. Luciferase activities were normalized by both GFP fluorescence and protein concentration. **d** Wild type or STAT3 mutant TWIST1 promoter-luciferase constructs were transfected with or without MKL1 into HEK293 cells or EAhy926 cells. Luciferase activities were normalized by both GFP fluorescence and protein concentration. **e** Human vascular endothelial cells were transfected with siRNA targeting STAT3 or scrambled siRNA (SCR) followed by treatment with TGF-β. ChIP assays were performed with indicated antibodies. **f** Co-immunoprecipitation was performed with indicated antibodies using lysates extracted from human vascular endothelial cells. **g** Human vascular endothelial cells were treated with TGF-β for 72 h. Cytoplasmic/nuclear proteins were extracted and blotted for MKL1 and STAT3. **h** Human vascular endothelial cells were treated with TGF-β for 72 h. Re-ChIP assays were performed with indicated antibodies.

Gene expression profiling indicated that one of the key EndMT regulators, Twist1, was significantly down-regulated in LSECs isolated from ecKO^m/m^ BDL mice compared to WT BDL mice (Fig. 4a); by comparison, Snail, Slug, or Zeb1 was not altered by MKL1 deficiency. Twist1 expression was induced by TGF-β in HVECs, but MKL1 silencing abrogated Twist1 induction (Fig. 4b). Similarly, CCG treatment abolished Twist1 induction by TGF-β in HVECs (Fig. S6A, B). CCG administration also resulted in a decrease in Twist1 expression in LSECs in mice subjected to the BDL procedure (Fig. S6C) or CCl_4_ injection (Fig. S6D) compared to the vehicle control.

We then transfected into HEK293 cells or endothelial cells Twist1 promoter-luciferase constructs of various lengths: MKL1 over-expression activated the Twist1 promoters until the deletion extended beyond −186 relative to the transcription start site (Fig. 4c). A STAT3 binding site has been identified between −186 and −105^25^; mutagenesis of this site abrogated the activation of the Twist1 promoter by MKL1 (Fig. 4d), suggesting that STAT3 might be responsible for recruiting MKL1 to the Twist1 promoter. ChIP assay showed that MKL1 occupancy on the proximal Twist 1 promoter, but not the Gapdh promoter, was enhanced by TGF-β treatment; STAT3 silencing (Fig. S2B for knockdown efficiency) severely compromised MKL1 binding (Fig. 4e). Co-immunoprecipitation assay showed that MKL1 and STAT3 formed a complex in endothelial cells (Fig. 4f). TGF−β treatment promoted the accumulation of both MKL1 and STAT3 in the nucleus (Fig. 4g). Of importance, TGF-β treatment enhanced the interaction between MKL1 and STAT3 on the Twist1 promoter (Fig. 4h). We conclude that MKL1 presumably contributes to EndMT by activating Twist1 transcription.

### STAT3 inhibition attenuates liver fibrosis in mice

**Fig. 5:**
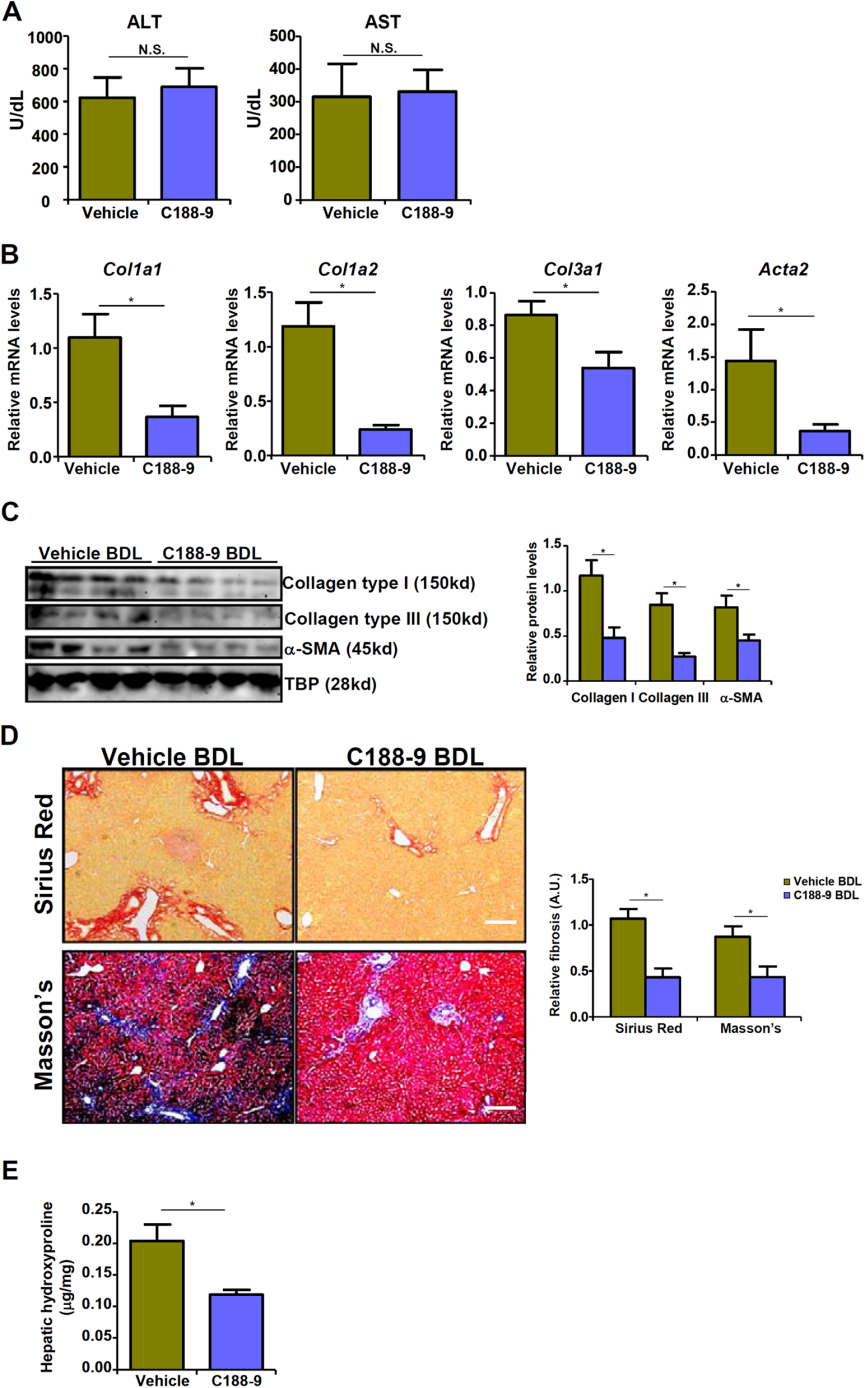
STAT3 inhibition attenuates liver fibrosis in mice. C57/BL6 mice were subjected to the BDL procedure. Following the surgery, the mice were injected peritoneally with C188-9 as described in Methods. **a** Plasma ALT levels and AST levels. Expression levels of pro-fibrogenic genes were examined by qPCR (**b**) and western (**c**). **d** Picrosirius red and Masson’s trichrome stainings. **e** Hepatic hydroxylproline levels. *N* = 6–8 mice for each group.

Now that STAT3 appeared to be essential in recruiting MKL1 to activate Twist1 transcription, we tested the hypothesis that STAT3 inhibition might serve to attenuate liver fibrosis. STAT3 inhibition by a small-molecule compound C188-9^26^ dampened TGF-β induced EndMT in HVECs in a dose-dependent manner (Fig. S7A, B). Attenuation of EndMT by C188-9 correlated with a decrease in MKL1 recruitment to the Twist1 promoter (Fig. S7C). Administration of C188-9 in mice did not significantly alter liver injury after the BDL procedure (Fig. 5a). Expression of pro-fibrogenic genes including collagen type I, collagen type III, and α-SMA, however, were collectively down-regulated by C188-9 (Fig. 5b, c). Additional evidence was provided by picrosirius red and Masson’s stainings (Fig. 5d) as well as hepatic hydroxylproline quantifications (Fig. 5e), which all pointed to a significant reduction in liver fibrosis as result of STAT3 inhibition.

### TWIST1 inhibition attenuates liver fibrosis in mice

**Fig. 6:**
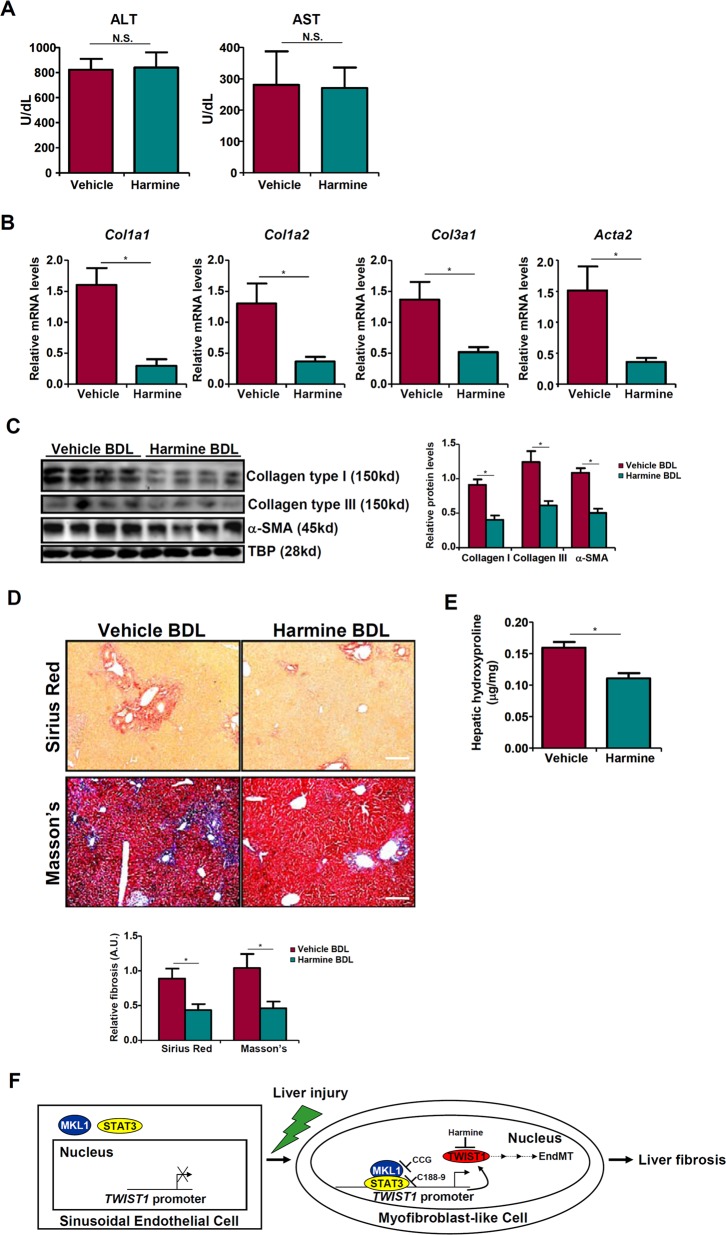
TWIST1 inhibition attenuates liver fibrosis in mice. C57/BL6 mice were subjected to the BDL procedure. Following the surgery, the mice were injected peritoneally with harmine as described in Methods. **a** Plasma ALT levels and AST levels. Expression levels of pro-fibrogenic genes were examined by qPCR (**b**) and western (**c**). **d** Picrosirius red and Masson’s trichrome stainings. **e** Hepatic hydroxylproline levels. *N* = 6–8 mice for each group. **f** A schematic model.

We finally examined whether pharmaceutical inhibition of TWIST1, the end-product of the STAT3-MKL1 transcriptional axis, would be sufficient to attenuate EndMT and liver fibrosis. Treatment with a small-molecule TWIST1 inhibitor harmine^27^ in HVECs partially alleviated the repression of *PECAM1* and *CDH5* and the activation of *COL1A2* and *ACTA2* under the influence of TGF-β (Fig. S8). Next, C57/BL6 mice were subjected to the BDL procedure to induce liver fibrosis, after which the mice were injected with harmine or vehicle daily for the duration of the experiment. As shown in Fig. 6a, harmine administration did not significantly alter liver injury. Harmine injection significantly decreased the expression of pro-fibrogenic genes in the liver (Fig. 6b, c). Harmine also diminished the accumulation of extracellular matrix proteins in the liver as evidenced by picrosirius red and Masson’s trichrome stainings (Fig. 6d) and hepatic hydroxylproline quantification (Fig. 6e). These data all support an indispensable role for TWIST1 in EndMT and liver fibrosis.

## Discussion

Myofibroblast activation is considered a paradigm underlying tissue fibrosis. Endothelial cells undergoing EndMT contribute to myofibroblast expansion and liver fibrosis^8,9^. Here we delineate a novel pathway underlying TGF-β induced EndMT and liver fibrosis in which the transcriptional modulator MKL1, in cooperation with the transcription factor STAT3 to activate TWIST1 transcription. More importantly, our data reveal that pharmaceutical inhibition of MKL1, STAT3, or TWIST1 using small-molecule compounds was paralleled by suppression of EndMT and attenuation of liver fibrosis (Fig. 6f), thus laying out the groundwork for the development of novel therapeutics to treat end-stage liver diseases.

MKL1 is ubiquitously expressed in different cell types in the liver. We have previously reported that systemic MKL1 deficiency in mice ameliorates both liver injury and liver fibrosis^20,21^. Of interest, although our previous reports using cultured hepatic stellate cells (HSCs) and portal fibroblast cells (PFCs) unequivocally demonstrate that MKL1 deficiency blocked the trans-differentiation of HSCs^21^ and PFCs^20^ into mature myofibroblasts, it remains untested whether genetic ablation of MKL1 specifically from either HSCs or PFCs would be sufficient to stall liver fibrosis in vivo. In contrast, endothelial-specific MKL1 deficiency retarded liver fibrosis without influencing liver injury (Fig. 1a and data not shown), suggesting that MKL1 possesses cell-specific roles regulating liver pathophysiology. Indeed, we have shown earlier that MKL1 promotes cardiac hypertrophy in non-cell-autonomous manner via activating endothelial cell-derived endothelin production^28^. More recently, we have reported that MKL1 regulates ischemia-reperfusion induced cardiomyocyte injury in a similar fashion by regulating macrophage-derived reactive oxygen species^23^. These observations altogether allude to a model wherein MKL1 regulates liver fibrosis in a highly cell-specific manner and its role as a driver of EndMT-associated fibrosis can be dissected from its role as a promoter of liver injury.

Several outstanding questions remain unanswered that await further investigation. First, although we focused on EndMT as a readout to gauge the contribution of endothelial MKL1 to liver fibrosis, other MKL1-dependent endothelial functions cannot be overlooked. For instance, LSEC-derived NO helps maintain the quiescent phenotype of HSCs thus preventing trans-differentiation of HSCs into myofibroblasts^29^. We have demonstrated previously that MKL1 is a transcriptional repressor of eNOS in endothelial cells thus limiting the availability of NO^30^. Therefore, it is reasonable to postulate that MKL1 deficiency in endothelial may lead to increased synthesis of NO and consequently decelerated myofibroblast activation. RNA-seq analysis would help evaluate the impact of MKL1 deficiency on endothelial transcriptome and unveil potential mechanisms underlying liver fibrosis. Second, our data suggest that MKL1 specifically regulated TWIST1 transcription in LSECs (Fig. 3a). Other transcription factors of the same family, including Snail^31^ and Slug^12^, have been implicated in TGF-β induced EndMT though none have been directly reported to regulate liver fibrosis. Morita et al. have shown that MKL1 directly activates Slug transcription to promote TGF-β induced epithelial-mesenchymal transition in renal tubular epithelial cells^32^. It is possible that MKL1 may contribute to TGF-β induced EndMT by differentially regulating Snail family of proteins in a tissue/context-dependent manner although this hypothesis needs further verification preferentially with endothelial-specific knockout animal models.

In summary, our observations argue for a role in EndMT and liver fibrosis for the STAT3-MKL1-TWIST1 axis. Since EndMT is hailed as a key driving force behind fibrotic diseases^33^, our data likely have far more broad implications than summarized here. Rigorous validations of the current working model (Fig. 6f) using additional animal models will hopefully pave the way for screening more effective small-molecule compounds that target this axis to treat liver fibrosis.

## Materials and methods

### Animals

All animal experiments were reviewed and approved by the Intramural Ethics Committee on Humane Treatment of Experimental Animals. All mice were bred at the Nanjing Biomedical Research Institute of Nanjing University (NBRI). Endothelial-specific deletion of MKL1 was achieved by crossing the *Mkl1*^f/f^ strain^23^ to the *Cdh5*-Cre strain^34^. Liver fibrosis was induced by bile duct ligation (BDL) or CCl_4_ injection (1.0 mL/kg body weight as 50%, vol/vol, weekly for 6 weeks)^20^. In certain experiments, the mice were injected peritoneally the MKL1 inhibitor CCG-1423 (1 mg/kg, Selleck), the STAT3 inhibitor C188-9 (50 mg/kg, Selleck), or the TWIST1 inhibitor harmine (10 mg/kg, Selleck) daily after the BDL procedure.

### Cell culture, plasmids, transient transfection, and reporter assay

HEK293 cells (ATCC) and immortalized human umbilical endothelial cells (EAhy926, ATCC) were maintained in DMEM supplemented with 10% FBS^35^. Primary human vascular endothelial cells (HVEC, Life Technologies) were maintained in Media 231 with supplements provided by the vendor^36^; at least three different batches of cells were used. Primary sinusoidal endothelial cells were isolated as previously described^37^. Briefly, the mice were anesthetized with isoflurane. Following perfusion and digestion, the liver suspension was passed through a 70-µm cell strainer. The non-parenchymal cells were isolated by density gradient centrifugation. LSECs were further purified by selective adherence for exactly 8 min. Expression constructs for MKL1^38^ and TWIST1 promoter-luciferase constructs^25,39^ have been described previously. Small interfering RNAs were purchased from Dharmacon. Transient transfection was performed with Lipofectamine 2000. Cells were harvested 48 h after transfection and reporter activity was measured using a luciferase reporter assay system (Promega).

### Protein extraction, immunoprecipitation, and western blot

Whole-cell lysates were obtained by re-suspending cell pellets in RIPA buffer (50 mM Tris pH7.4, 150 mM NaCl, 1% Triton X-100) with freshly added protease inhibitor (Roche) as previously described^24^. Nuclear lysates were prepared with the NE-PER Kit (Pierce) following manufacturer’s recommendation. Western blot analyses were performed with anti-MKL1 (Santa Cruz, sc-32909), anti-collagen type I (Rockland, 600-401-103), anti-α-SMA (Abcam, ab5694), anti-STAT3 (Cell Signaling Technology, 9132), anti-TWIST1 (Abcam, ab50887), anti-VE-Cadherin (Cell Signaling Technology, 2158), anti-PECAM1 (Proteintech, 11265-1), anti-α-tubulin (Sigma, T5168), anti-Lamin B (Santa Cruz, sc-6216), and anti-β-actin (Sigma, A2228) antibodies. All experiments were repeated three times.

### RNA isolation and real-time PCR

RNA was extracted with the RNeasy RNA isolation kit (Qiagen) as previously described^40–44^. Reverse transcriptase reactions were performed using a SuperScript First-strand Synthesis System (Invitrogen). Real-time PCR reactions were performed on an ABI Prism 7500 system. Primers and Taqman probes used for real-time reactions were purchased from Sangon Biotech (Shanghai, China).

### Chromatin immunoprecipitation

Chromatin Immunoprecipitation (ChIP) assays were performed essentially as described before^45–49^. In brief, chromatin in control and treated cells were cross-linked with 1% formaldehyde. Cells were incubated in lysis buffer (150 mM NaCl, 25 mM Tris pH 7.5, 1% Triton X-100, 0.1% SDS, 0.5% deoxycholate) supplemented with protease inhibitor tablet and PMSF. DNA was fragmented into ∼200 bp pieces using a Branson 250 sonicator. Aliquots of lysates containing 200 μg of protein were used for each immunoprecipitation reaction with anti-MKL1 (Santa Cruz, sc-32909), anti-STAT3 (Cell Signaling Technology, 9132), or pre-immune IgG. For re-ChIP, immune complexes were eluted with the elution buffer (1% SDS, 100 mM NaCO_3_), diluted with the re-ChIP buffer (1% Triton X-100, 2 mM EDTA, 150 mM NaCl, 20 mM Tris pH 8.1), and subject to immunoprecipitation with a second antibody of interest.

### Histology

Histologic analyses were performed essentially as described before^38,50–52^. Briefly, paraffin-embedded sections were stained with picrosirius red (Sigma-Aldrich) orMasson trichrome (Sigma-Aldrich) according to standard procedures. Pictures were taken using an Olympus IX-70 microscope (Olympus, Tokyo, Japan).

### Immunofluorescence microscopy

Immunofluorescence was performed as previously described^53–56^. Liver tissues were fixed with 4% formaldehyde, permeabilized with TBST (.25% Triton X-100, 150 mM NaCl, 50 mM Tris pH7.4), blocked with 5% BSA, and incubated with indicated primary antibodies overnight. After several washes with PBS, cells were incubated with FITC-labeled secondary antibodies (Jackson) for 30 min. DAPI (Sigma) was added and incubated with cells for 5 min prior to observation. Immunofluorescence was visualized on a co-focal microscope (LSM 710, Zeiss).

### Statistical analysis

One-way ANOVA with post-hoc Scheff´e analyses were performed by SPSS software (IBM SPSS v18.0, Chicago, IL, USA). Unless otherwise specified, values of *p* < 0.05 were considered statistically significant.

## Supplementary information

suppplementary figure legends

Fig.S1

Fig.S2

Fig.S3

Fig.S4

Fig.S5

Fig.S6

Fig.S7

Fig.S8
