# Supplementary material for: MKL1 promotes endothelial-to-mesenchymal transition and liver fibrosis by activating TWIST1 transcription
Source: Cell Death Dis. 2019 Nov 27;10(12):899. doi: 10.1038/s41419-019-2101-4 (PMC6881349; doi:10.1038/s41419-019-2101-4)
Supplement: Supplementary file 1 — suppplementary figure legends [file 41419_2019_2101_MOESM1_ESM.doc]

**Li ZL *et al*: *MKL1 promotes endothelial-mesenchymal transition and liver fibrosis by activating TWIST1 transcription***

**Online supplementary material**

**Supplementary figure legends**

**Fig.S1**: ***Verification of endothelial deletion of MKL1***. Liver sections from WT and ecKO^m/m^ mice were stained with CD31 (green) and MKL1 (red). Scale bar, 50μm.

**Fig.S2**: ***Verification of siRNA knockdown efficiencies.*** (**A**) HVECs were transfected with siRNA targeting MKL1 (siMKL1) or scrambled siRNA (SCR). MKL1 expression was examined by qPCR and Western. (**B**) HVECs were transfected with siRNA targeting STAT3 (siSTAT3) or scrambled siRNA (SCR). STAT3 expression was examined by qPCR and Western.

**Fig.S3**: ***MKL1 knockdown attenuates EndMT***. (**A,B**) Human vascular endothelial cells were transfected with a second pair of siRNAs targeting MKL1 or scrambled siRNA (SCR) followed by treatment with TGF-β. Gene expression levels were examined by qPCR and Western.

**Fig.S4**: ***CCG-1423 administration attenuates BDL-induced liver fibrosis in mice*.** Liver fibrosis was induced in C57/B6 mice by BDL followed by CCG (1mg/kg/d) injection as described in Methods. (**A**)Expression levels of pro-fibrogenic genes were examined by qPCR. (**B**) Picrosirius red and Masson’s trichrome stainings. (**C**) LSECs were isolated and gene expression was examined by qPCR. N=3~6 mice for each group.

**Fig.S5**: ***CCG-1423 administration attenuates CCl_4_-induced liver fibrosis in mice*.** Liver fibrosis was induced in C57/B6 mice by CCl_4_ followed by CCG (1mg/kg/d) injection as described in Methods. (**A**) Expression levels of pro-fibrogenic genes were examined by qPCR. (**B**) Picrosirius red and Masson’s trichrome stainings. (**C**) LSECs were isolated and gene expression was examined by qPCR. N=3~6 mice for each group.

**Fig.S6**: ***CCG-1423 represses TWIST1 expression in vitro and in vivo*.** (**A, B**) Human vascular endothelial cells were treated with TGF-β in the presence or absence of CCG-1423. TWIST1 expression levels were examined by qPCR and Western. (**C**) Liver fibrosis was induced in C57/B6 mice by BDL followed by CCG (1mg/kg/d) injection as described in Methods. TWIST1 expression levels were examined by qPCR. (**D**) Liver fibrosis was induced in C57/B6 mice by CCl_4_ followed by CCG (1mg/kg/d) injection as described in Methods. TWIST1 expression levels were examined by qPCR.

**Fig.S7: *STAT3 inhibition by C188-9 attenuates TGF-β induced EndMT in HVECs***. Human vascular endothelial cells were treated with TGF-β in the presence or absence of C188-9. Gene expression levels were examined by qPCR (**A**) and Western (**B**). ChIP assay was performed with anti-MKL1 (**C**).

**Fig.S8: *TWIST1 inhibition by harmine attenuates TGF-β induced EndMT in HVECs***. Human vascular endothelial cells were treated with TGF-β in the presence or absence of harmine. Gene expression levels were examined by qPCR (**A**) and Western (**B**).
