# Supplementary figures and images for: MKL1 promotes endothelial-to-mesenchymal transition and liver fibrosis by activating TWIST1 transcription
Source: Cell Death Dis. 2019 Nov 27;10(12):899. doi: 10.1038/s41419-019-2101-4 (PMC6881349; doi:10.1038/s41419-019-2101-4)

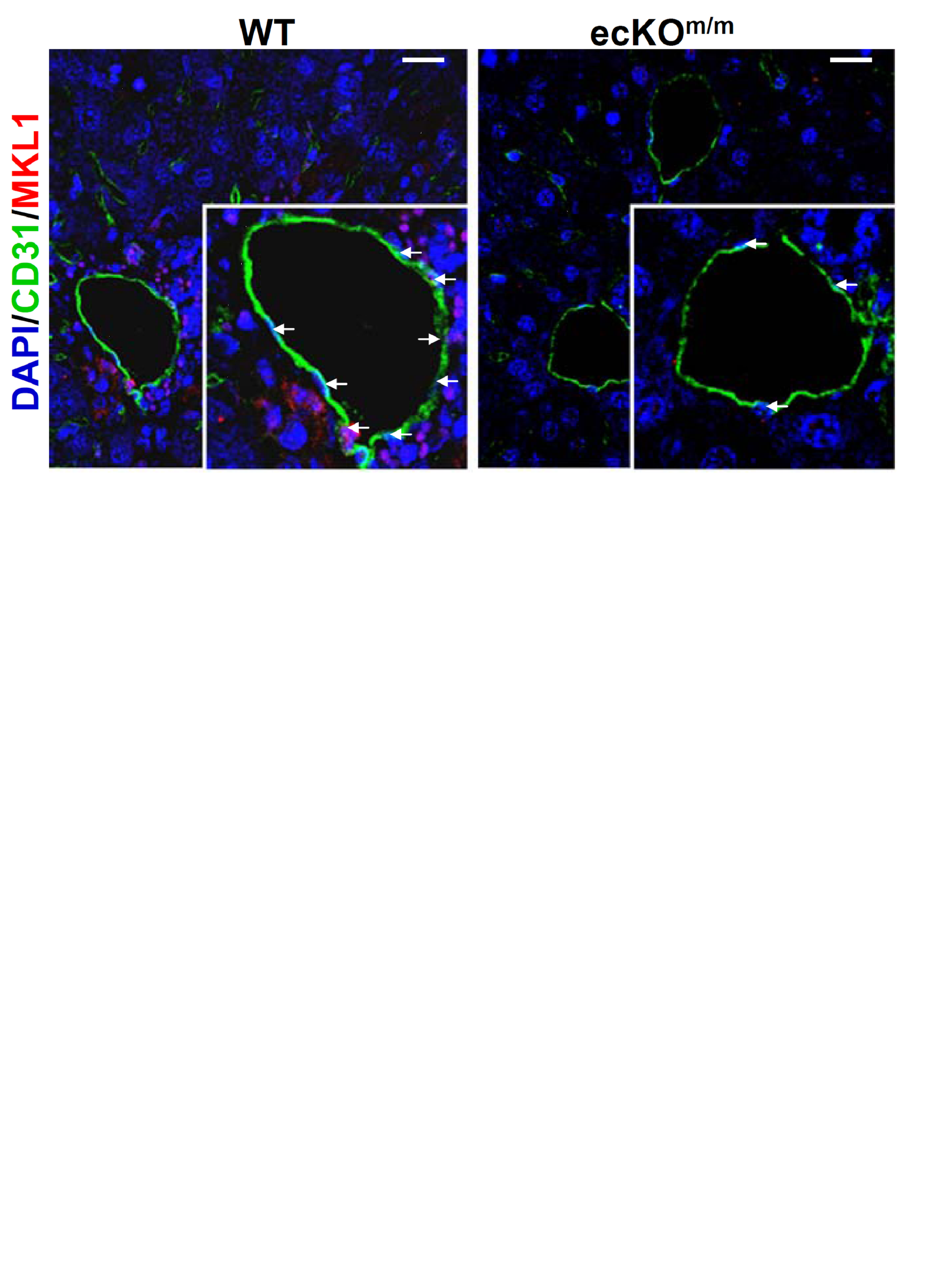

Supplement: Supplementary file 2 — Fig.S1 [file 41419_2019_2101_MOESM2_ESM.tif]

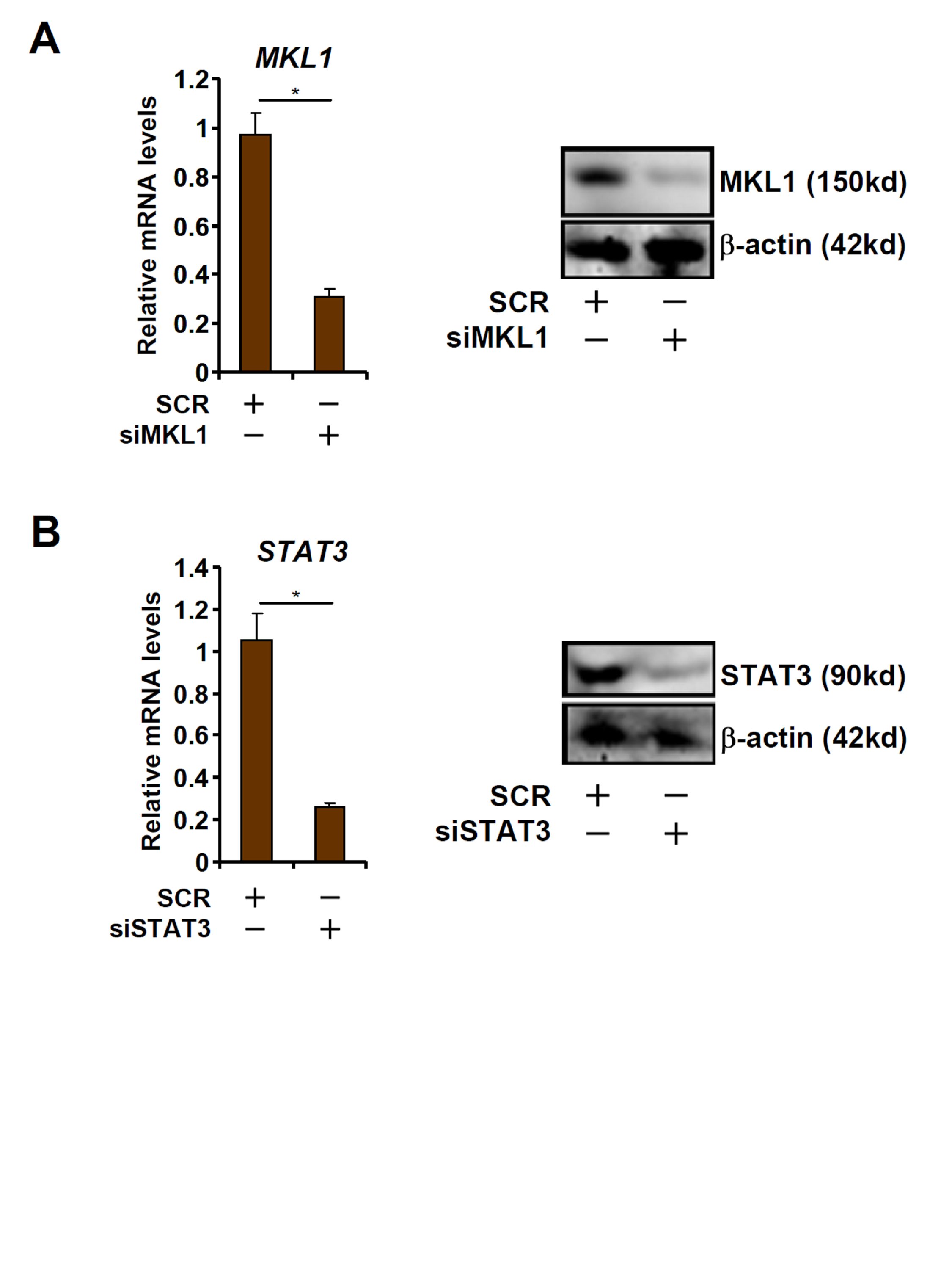

Supplement: Supplementary file 3 — Fig.S2 [file 41419_2019_2101_MOESM3_ESM.tif]

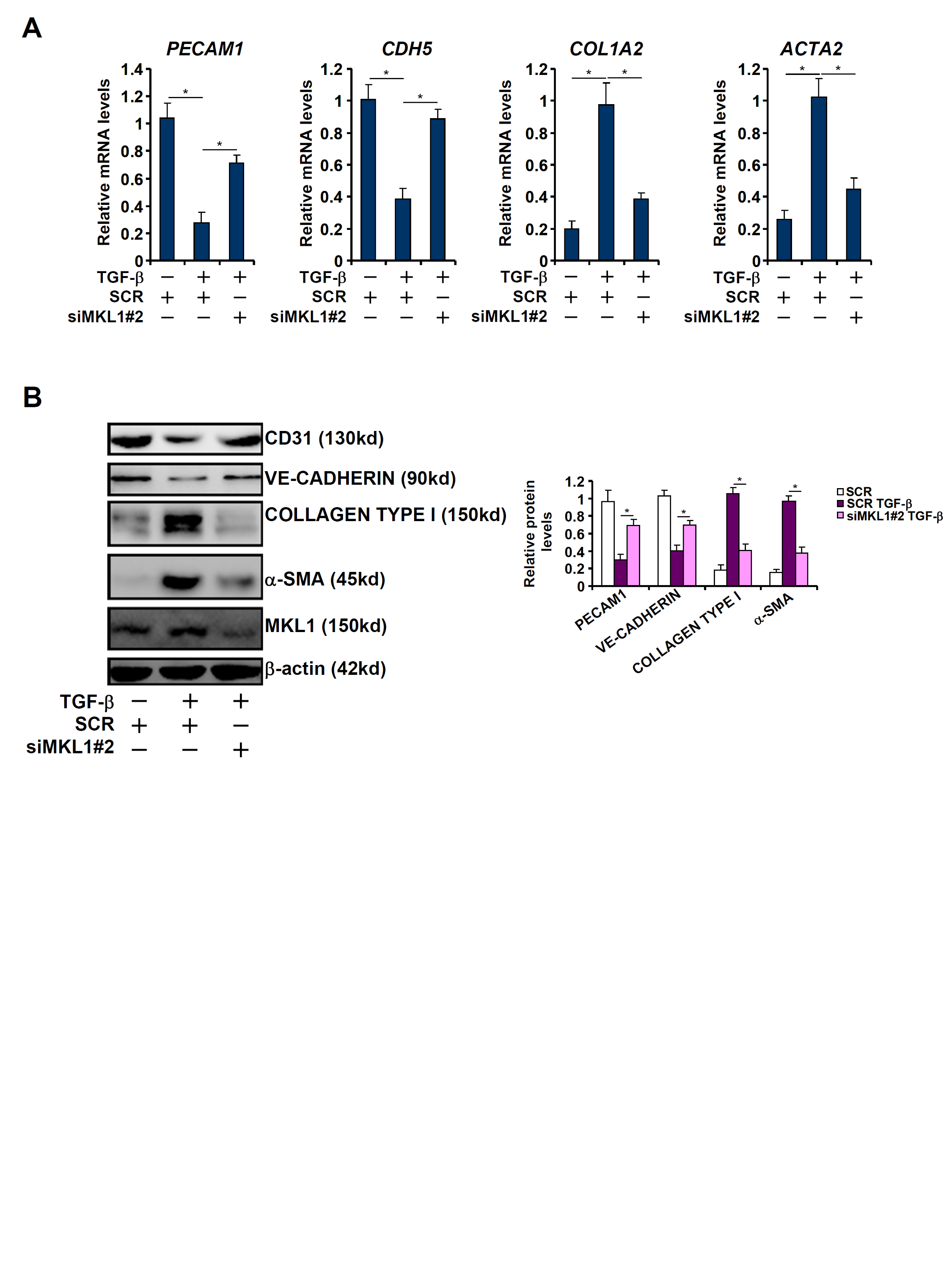

Supplement: Supplementary file 4 — Fig.S3 [file 41419_2019_2101_MOESM4_ESM.tif]

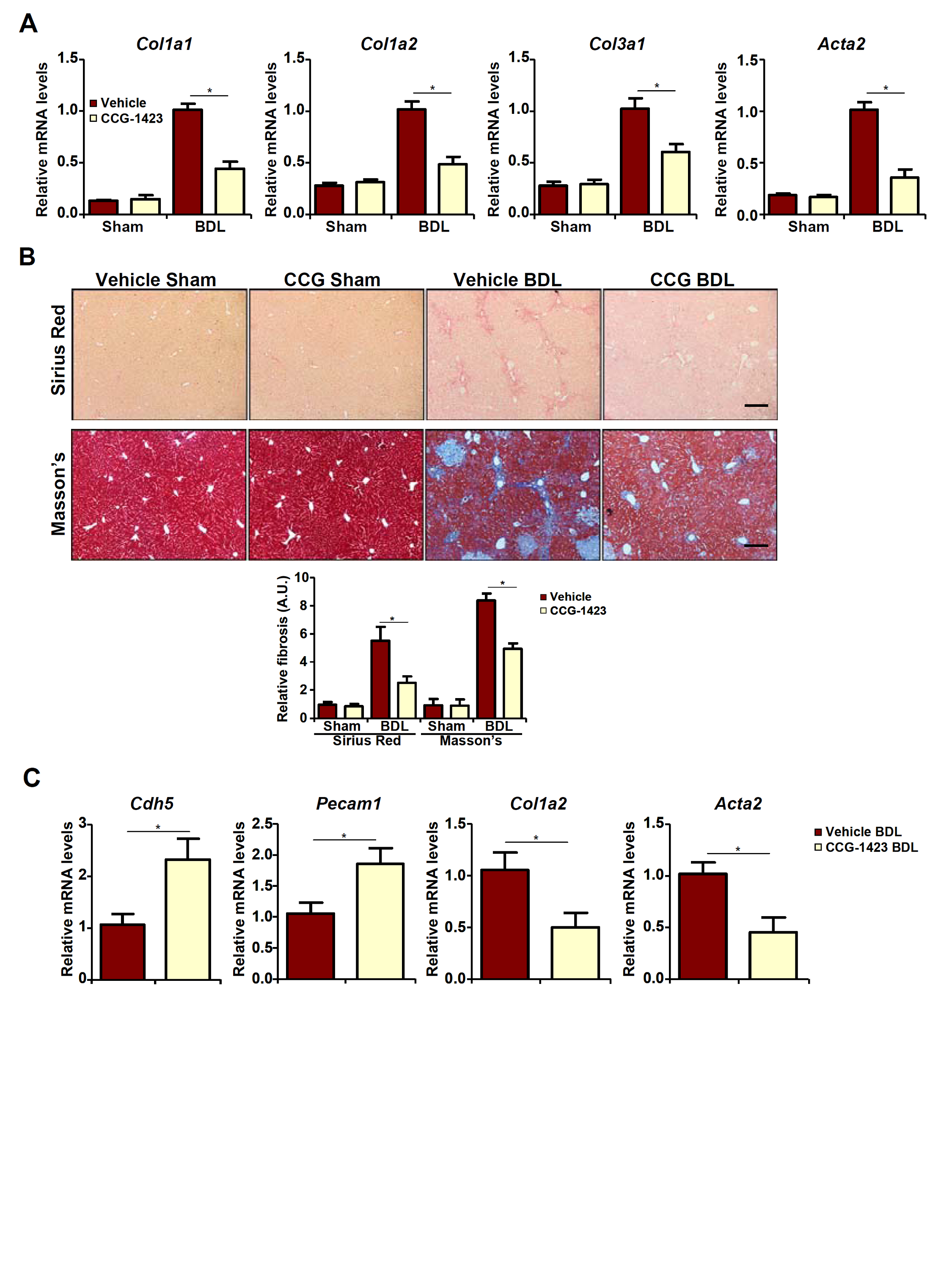

Supplement: Supplementary file 5 — Fig.S4 [file 41419_2019_2101_MOESM5_ESM.tif]

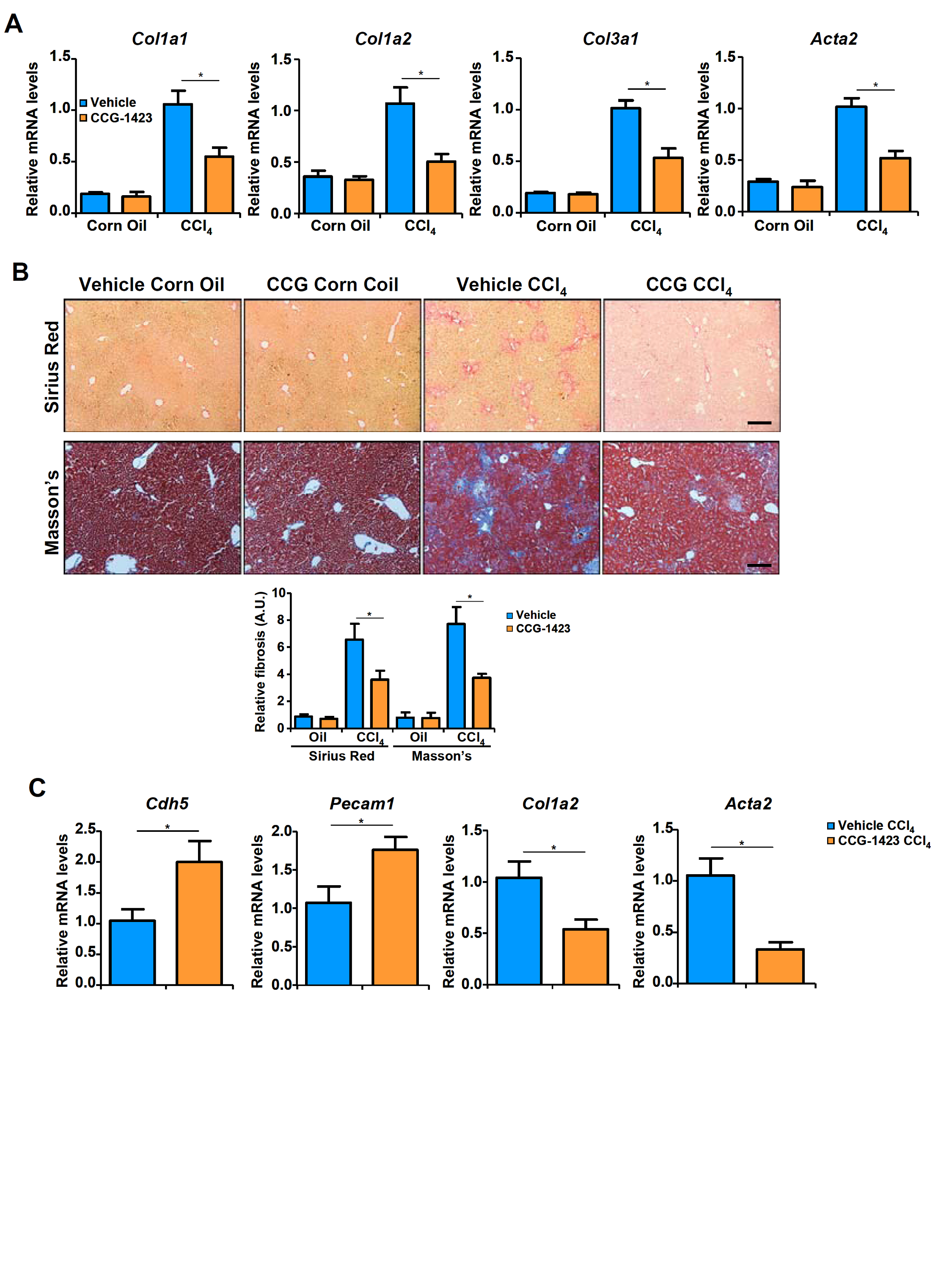

Supplement: Supplementary file 6 — Fig.S5 [file 41419_2019_2101_MOESM6_ESM.tif]

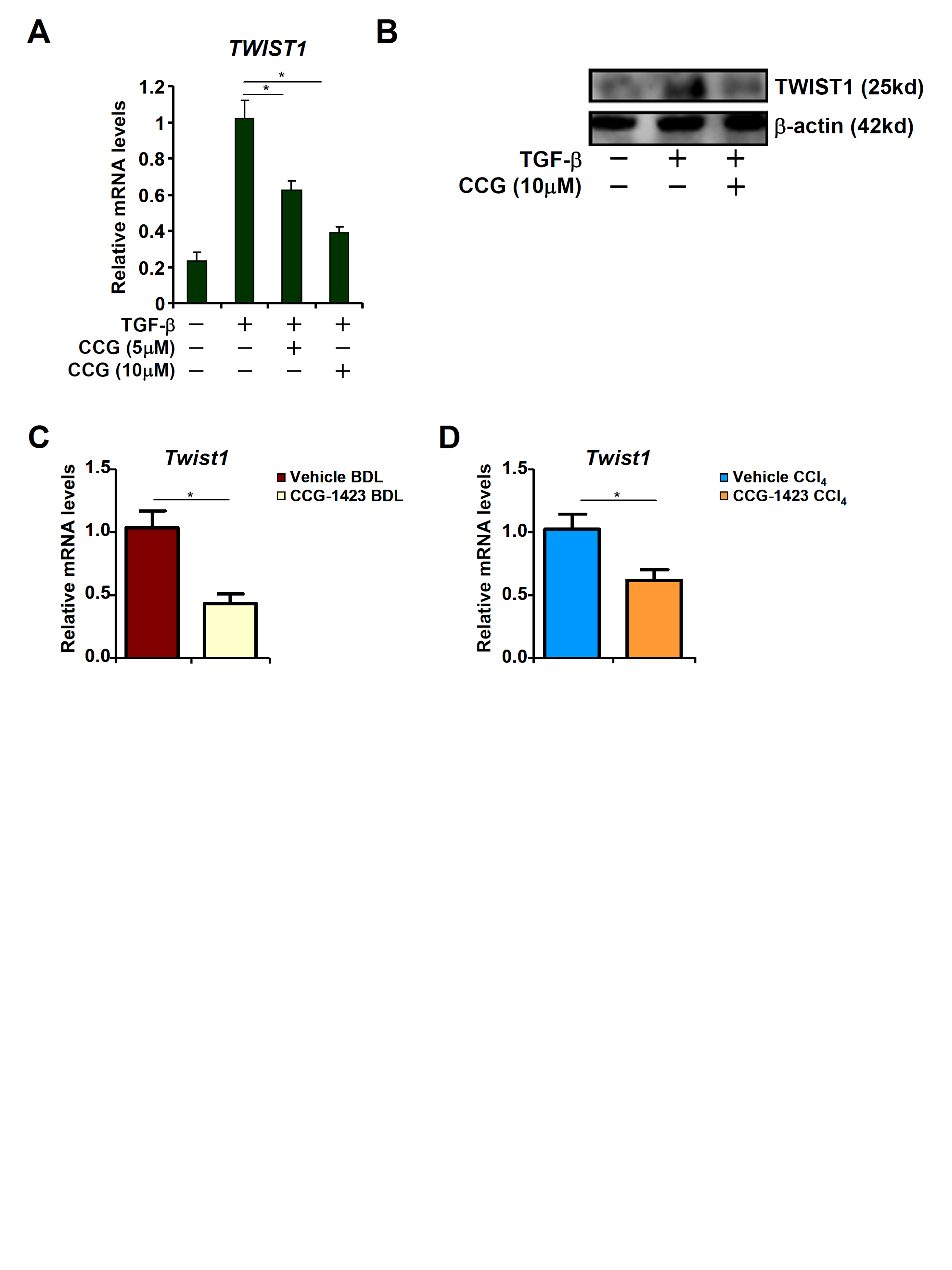

Supplement: Supplementary file 7 — Fig.S6 [file 41419_2019_2101_MOESM7_ESM.tif]

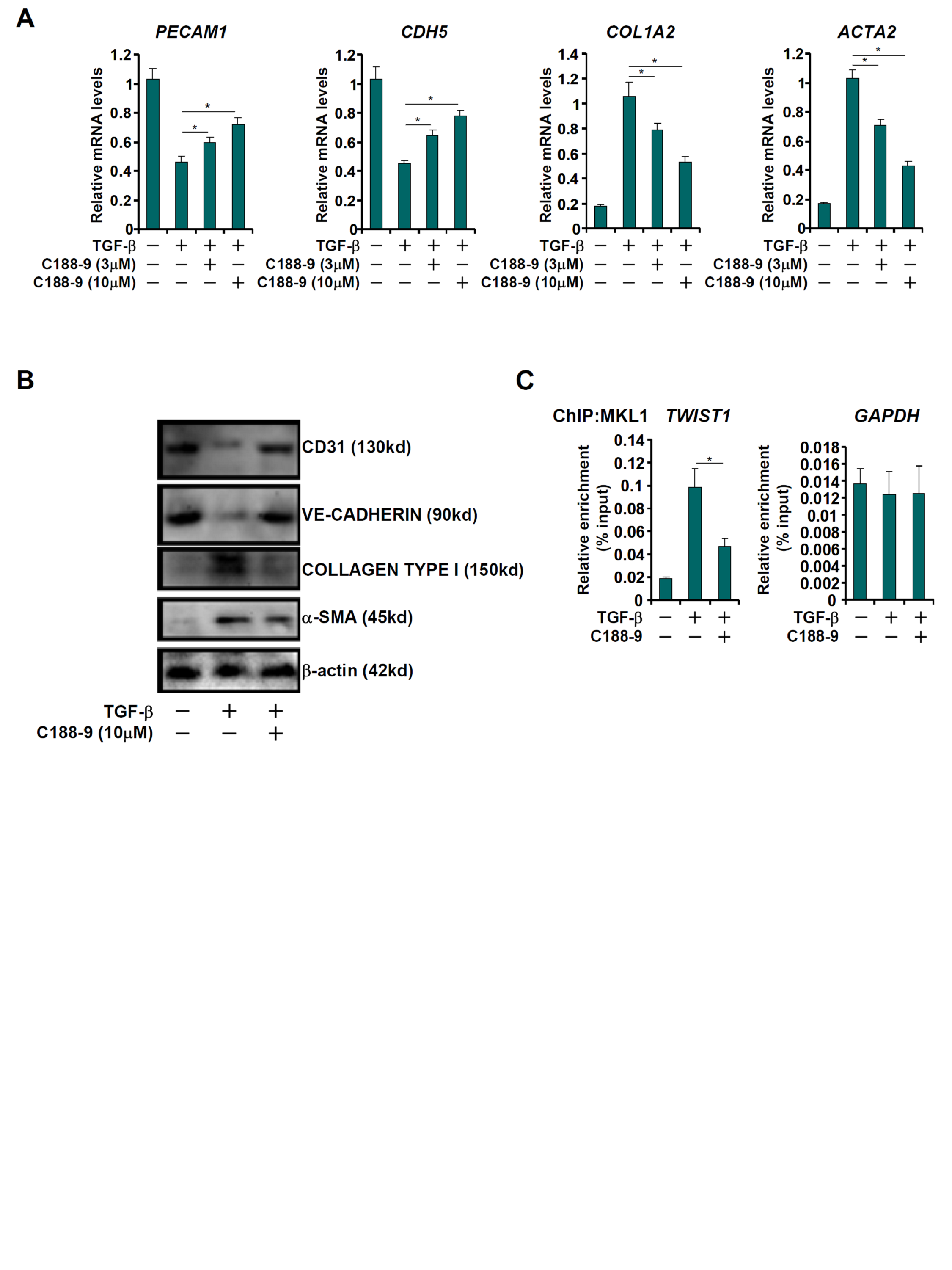

Supplement: Supplementary file 8 — Fig.S7 [file 41419_2019_2101_MOESM8_ESM.tif]

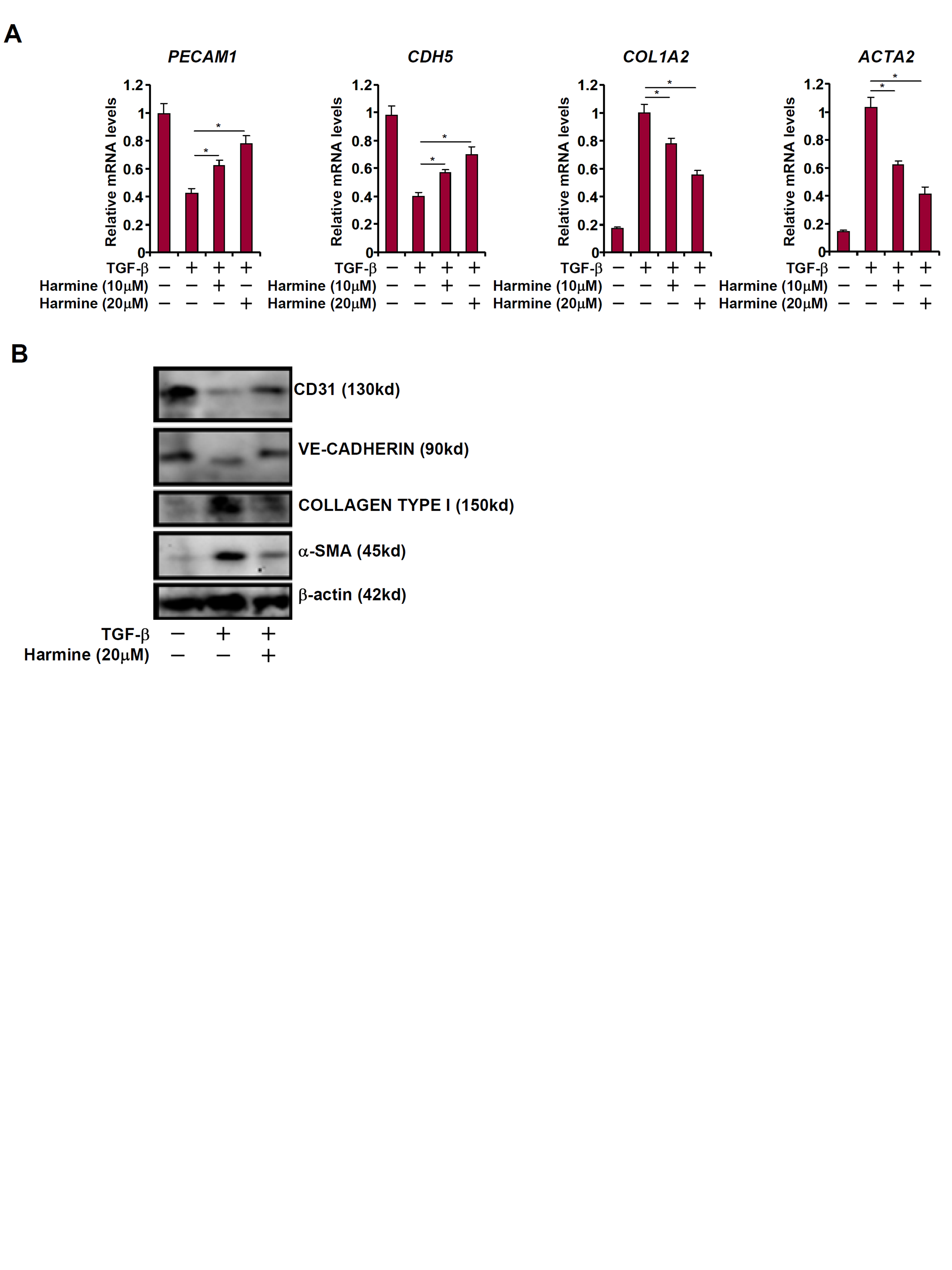

Supplement: Supplementary file 9 — Fig.S8 [file 41419_2019_2101_MOESM9_ESM.tif]
